# Judgment in Mild Cognitive Impairment and Alzheimer's disease

**DOI:** 10.1590/S1980-57642011DN05040007

**Published:** 2011

**Authors:** Patrícia Helena Figueirêdo Vale Capucho, Sonia Maria Dozzi Brucki

**Affiliations:** 1 Cognitive and Behavioral Neurology Group of Clínicas Hospital of the University of São Paulo School of Medicine (FMUSP), Referral Center for Cognitive Disorders (CEREDIC) of the FMUSP, São Paulo SP, Brazil.

**Keywords:** judgment, dementia, Alzheimer's disease, Mild Cognitive Impairment

## Abstract

Judgment is the capacity to make decisions after considering available information, contextual factors, possible solutions and probable outcomes. Our aim was to investigate previous research studies regarding assessment of judgment in older adults with different degrees of cognitive impairment. To this end, a search of Pubmed and Lilacs electronic databases for studies published from January 1990 until August 2011 in English, Spanish and Portuguese was carried out. The terms used were "judgment" combined with the terms "dementia" or "Mild Cognitive Impairment" (MCI) or "Alzheimer's disease" (AD). Some studies showed that MCI and AD patients had impaired judgment. There is a lack of specific methods to measure judgment capacity, and data on judgment abilities in older adults with MCI and dementia are scarce. No studies with specific measures of judgment capacity in other dementias were found.

## Abstract (pt)

Julgamento é a capacidade de tomar decisões após consideração sobre informações disponíveis, fatores contextuais, soluções possíveis e resultados prováveis. Nosso objetivo foi investigar estudos de pesquisas prévias sobre avaliação de julgamento em adultos mais velhos com diferentes graus de prejuízo cognitivo. Nós realizamos uma pesquisa nas bases eletrônicas de dados Pubmed e Lilacs, de estudos publicados de Janeiro de 1990 até Agosto de 2011, em Inglês, Espanhol e Português. Os termos usados foram "julgamento" combinado com os termos "demência" ou "Comprometimento Cognitivo Leve" (CCL) ou "doença de Alzheimer" (DA). Alguns estudos mostraram que pacientes com CCL e DA tem comprometimento na capacidade de julgamento. Há uma carência de métodos específicos para avaliar julgamento, e dados sobre habilidades de julgamento em adultos mais velhos com CCL e demência são escassos. Não foram encontrados estudos com medidas específicas da capacidade de julgamento em outras demências.

## Introduction

Judgment can be defined as the capacity to make decisions after careful consideration of available information, contextual factors, possible solutions and probable outcomes. It is intimately linked with the process of problem solving and decision-making, and these terms are often used interchangeably in the neuropsychological literature.^1^

The conceptual template for a decision includes three components: courses of action, uncertain events and consequences. Decision-making refers to the entire process of choosing a course of action. Judgment refers to the components of the decision-making process that are concerned with assessing, estimating and inferring what events will occur and what the decision-maker's evaluative reactions to those outcomes will be.^2^

From this perspective, judgment is more an evaluative process (the act of settling on a decision/solution after going through the stages of active problem solving) and can be considered one of the last stages of active problem solving. Stating that a person has "bad judgment" means that this person made a poor decision after considering the information/context available.^1^

Thus, it can be understood that the outcomes of the decisions are based on judgment. For the execution of good judgment it is necessary to attempt to comprehend a situation, to create adequate strategies to approach a problem, identify appropriate goals, make choices between one idea or another, evaluate potential consequences of different courses of action, inhibit inappropriate responses, initiate purposeful behaviors and monitor the effects of a chosen solution.^2-6^

From a neuropsychological perspective, the concept of judgment involves many cognitive aspects including memory, language, sustained attention and reasoning^6,7^ but especially engages the executive functions.^8,9^ Beyond cognitive functions, judgment is also involved with emotional aspects^2,10,11^ and social conventions.^8^

Executive function refers to the capacity to engage in complex and objective behaviors, which require working memory, planning, organization, sequencing and abstraction. People with deficits in executive functions can present poor judgment for many reasons, such as taking impulsive decisions, focusing on only one solution because of reduced mental flexibility or failing to consider long-term outcomes.^3,4,8^

Research on decision making seeks to investigate preferential choice and action and how individuals choose action to achieve labile and conflicting goals in an uncertain world. The assessment of decision-making problems is usually carried out through the use of tasks which lack ecological validity such as gambling tasks involving uncertain alternatives for ambiguous or risky conditions sharply partitioned into gains and losses.^12,13^

For problem-solving assessment, several traditional neuropsychological measures have been employed, usually with the aim of assessing executive functions.^14,15^ Instrumental Activities of Daily Living (IADL) are closely related to everyday problem solving^6^ and some studies use IADL scales (usually answered by informants) to describe problem solving abilities.^16,17^

However, these standardized measures do not constitute an adequate means of evaluating behavioral quality during daily judgment, a task requiring the subject to search and look for the information and determine its relevance before making a decision. Moreover, measures that are not ecological might underestimate the extent of everyday difficulties by providing cues and relevant information together, content which is often not available in real life.^18^

Some scales have been developed for assessing competence to consent to medical treatment or research, such as the MacArthur Competency Assessment Tool for Treatment,^19^ Capacity to Consent to Treatment Instrument,^20^ Hopkins Competency Assessment Test,^21^ Aid to Capacity Evaluation^22^ and Assessment of the Capacity for Everyday Decision Making.^23^ These measures provide useful information about factors related to medical care decisions. However, they are specific to particular situations and are therefore not representative of general conditions. Patients who have impaired capacity to make decisions regarding their medical treatment do not necessarily lack capacity to make judgments and decisions concerning other aspects of their life.^24^

There are some tools for the measurement of multidimensional constructs that overlap with judgment (everyday problem solving, everyday decision making, social problem solving and practical intelligence) such as the Predicaments Task,^18^ Everyday Problem Solving Inventory,^25^ Reflective Judgment Dilemmas,^26^ Practical Problems Test,^27^ Everyday Cognition Battery,^7^ Everyday Problems Test (EPT)^28^ and Everyday Problems Test for Cognitively Challenged Elderly (EPCCE).^29^ These tests, however, were developed primarily for research purposes, lacking detailed information about their psychometric properties and are not routinely utilized by neuropsychologists.^1^ The EPT and EPCCE were studied in older adults with cognitive impairments and will be described in the sections that follow.

The aim of this report was to provide a review on the available instruments to specifically evaluate judgment capacity in older adults with different degrees of cognitive impairment.

## Methods

A comprehensive literature search of PubMed and Lilacs databases including articles published in English, Spanish and Portuguese from January 1990 to August 2011 was carried out using the terms "judgment" combined with the terms "dementia" or "Mild Cognitive Impairment" or "Alzheimer's disease". Reports with abstracts were selected and reviews and meta-analysis also included.

The initial search retrieved 286 articles. Of these, 243 redundant items were excluded because they were found in more than one combination of terms (for example in the combination of Judgment and Dementia as well as in Judgment and Alzheimer's disease) or were related with other aspects of judgment, such as juridical judgment or legal competency, visual, sound or olfactory judgment and judgment of family members of the patients regarding their capacities. Out of the remaining 43 articles, a further 13 were excluded because they focused on problem solving and decision making evaluations in dementia of other etiologies (using traditional and low ecological measures or IADL scales answered by informants) without clearly distinguishing these processes from judgment. After reading the 30 remaining papers, there was the inclusion of an additional 17 articles cited in references and not identified in the search because they were not indexed on Pubmed and Lilacs, were published before 1990 or were more focused on problem solving than on judgment. Therefore, a total of 47 papers were included in this review.

The existing measures of judgment will be outlined and then the studies regarding judgment tests and related constructs in Mild Cognitive Impairments and dementia patients described, from earliest to the most recent publications.

### Measures of judgment

Research on judgment has been driven by analogies between perception and prediction and the central questions concerning the process by which as-yet unknown events, outcomes and consequences can be inferred. Measures of judgment have been neglected and require reliable measurement instruments.

The only standardized judgment tests for older people are the Judgment Questionnaire subtest of the Neurobehavioral Cognitive Status Exam (NCSE JQ),^30^ the Problem Solving Subscale of the Independent Living Scales (ILS)^31^ and the Judgment / Daily Living Subtest of the Neuropsychological Assessment Battery (NAB JDC).^32^

The NCSE JQ is designed to examine judgment by posing four problematic situations and by asking the person to describe what he or she would do in response. However, its manual offers minimal guidelines to assist the examiner in administration and scoring, provides little data to support interpretation and the normative data are sparse.^8^

The ILS has five subscales (Memory-orientation; Managing money; Managing home and transportation; Healthy and Safety; Social Adjustment) and two domains observed in factor analysis of the subscales. These domains are performance-information and problem solving. The problem-solving factor subscale comprises 33 items across all subscales that evaluate abstract reasoning and judgment required for daily living. An example sample item is, "What would you do if your lights and television cut out simultaneously?" The scales take 20 to 25 minutes to administer.^33^

The NAB is composed of 24 tests that comprise five modules: Attention, Language, Memory, Spatial and Executive Function (including judgment, planning, conceptualization, mental flexibility and verbal fluency). The Judgment Subtest is composed of 10 items. Several studies evaluating NAB performance in older adults are available^34,35^ but no studies specifically describing the Judgment Subtest in this population were found. However, the items deal predominantly with basic safety and hygiene issues and less with high-level judgment dilemmas.^5^

The three tests described above are part of larger batteries and have statistical limitations including insensitivity for detecting dementia.^1,8,36^

Rabin et al.,^1^ evaluated neuropsychologist's practices and perspectives regarding judgment assessment. The objectives were to evaluate the frequency of judgment assessment in neuropsychological evaluations to identify the instruments most frequently used, the profile of patients submitted to the test, and the need for additional measures. The participants were members of the International Neuropsychological Society and of the National Neuropsychological Academy, holding doctoral degrees, living in the United States or Canada. The tests most frequently used to assess judgment were: Comprehension-WAIS-III (39%), Wisconsin Card Sorting Test (WCST) (36%) and Similarities-WAIS-III (19%), NCSE JQ (14%) and NAB JDG (6%). The authors discussed that, the three most used measures (Comprehension, WCST and Similarities) were not developed to assess judgment per se, but to evaluate the capacity to deal with general problem solving and investigate basic aspects of safety and hygiene. Approximately 90% of research respondents stated that it is necessary to create additional specific measures for assessing judgment.

In response to the need for a relevant clinical measure of everyday judgment in older adults, Rabin et al.,^5^ developed the Test of Practical Judgment (TOP-J). This constitutes a 15-item open-ended questionnaire in which participants listen to brief scenarios about everyday problems and report aloud their proposed solutions. These scenarios are representative of the types of judgment problems faced by older adults and the issues are related to four content domains: safety, social/ethical, financial and medical. The respective responses are recorded verbatim and scored on a 4-point scale with higher values indicating better judgment. The instrument takes approximately 10 minutes to administer and score.

Rabin et al.^37^ used voxel-based morphometry (VBM) to analyze the relationship between regional gray matter (GM) density and judgment ability (assessed by TOP-J). Participants included 120 older adults at least 60 years of age, classified as having Alzheimer's disease (AD), Mild Cognitive Impairment (MCI), healthy controls without complaints (HC) and controls with normal cognition but with complaints (CC). The TOP-J scores correlated with GM density in the left inferior frontal gyrus and to a lesser extent in the frontal superior gyrus.

Borgos et al.^38^ observed that psychiatric patients, including schizophrenia, schizoaffective disorder, bipolar disorder, borderline personality disorder and past substance dependence had worse performance on the TOP-J compared to controls. Pickens et al.,^39^ reviewed the psychometric proprieties of 18 measures of executive functions for adults with and without cognitive impairments. Only the TOP-J demonstrated adequate indices of reliability and validity.

### Judgment in Mild Cognitive Impairment

There is a body of evidence suggesting that difficulties problem solving measured with IADL scales or traditional neuropsychological measures^16,17,40,41^ and decision making measured by gambling tasks^42^ can be observed in older adults with MCI.

The detection of decline in judgment capacity, as well as in daily functioning in these patients, depends on the sensitivity of the method of evaluation used to detect the alterations, in general subtle, suffered by these patients.^16^

With respect to measures of constructs that overlap with judgment, two studies investigating this topic were found and demonstrated that individuals with MCI have poorer performance in these abilities compared with controls. Burton et al.,^43^ evaluated 250 subjects (158 controls and 92 MCI) on the EPT (cited above). The EPT consists of printed stimuli related to medication use, meal preparation, telephone use, shopping, financial management, household management and transportation. Controls obtained significantly better scores than patients with single domain MCI and this latter group obtained significantly better scores than the multiple-domain MCI groups. The authors discussed that the findings may more strongly reflect changes in cognitive functioning than changes in functional abilities. In another study, Burton et al.,^44^ evaluated performance on the EPT in 304 subjects classified as cognitively intact and cognitively impaired no dementia. The impaired group had statistically significant lower scores.

Although impairments in constructs related to judgment are present in MCI, only one study on specific judgment ability was found in this population.^5^ Of the four measures of judgment described in this paper (NAB JDC, NCSE JQ, Problem Solving Subscale of ILS and TOP-J), performance in MCI was evaluated only by the TOP-J and the NCSE JQ (Table 1). No studies were found regarding the judgment tests NAB JDC and Problem Solving Subscale of the ILS in these patients. The TOP-J (but not the NCSE JQ) seems to be a reliable measure for differentiating MCI from Control subjects and AD patients.

**Table 1:** Studies of performance of MCI and dementia patients on tests of judgment*.

Study	Sample	Test	Results
Drane and Osato, 1997^36^	Controls and dementia defined by DSM-III-R without type specification.	NCSE JQ	No statistically significant difference in performance of the groups was found.
Woods et al., 2000^5^	40 controls and 95 AD divided into more severely impaired (MMSE<20) and high functioning.(MMSE ≥20)	NCSE JQ	A statistically significant difference between controls and more impaired AD was found, but not between controls and high functioning AD.
Baird, 2006^50^	83 older adults divided into normal cognition, borderline cognition, mild dementia, and moderate dementia.	ILS	Similar profiles between borderline impairment and mild dementia. Patients with moderate dementia had poorer performance and subjects with normal cognition had better scores on all subscales, including the Problem Solving Subscale.
Rabin et al., 2007^5^	26 AD, 34 MCI, 39 subjects with normal cognition with complaints (CC) and 35 controls without complaints (HC).	NCSE JQ and TOP-J	On NCSE JQ, no statistically significant difference between the groups was found. On TOP-J. No difference between CC and MCI was found. These groups had lower scores than HC and higher scores than AD.

* NCSE JQ: Judgment Questionnaire subtest of the Neurobehavioral Cognitive Status Exam; ILS: Independent Living Scales; TOP-J: Test of Practical Judgment.

### Judgment in dementia

Loss of judgment capacity is common in dementia, when cognitive functions allowing the use of purposeful behaviors progressively fail.^9,45^

Although some patients with dementia can perform routine activities and tasks adequately, the ability to solve more complex problems such as those found within work, social and domestic environments and in interpersonal relationships are usually affected^3^. Problem solving, measured by traditional neuropsychological tests or IADL scales^17,46^ and decision making, evaluated by gambling tasks^47,48^ are known to be affected in AD patients.

Willis et al.,^49^ evaluated the performance of 65 older adults with mild to moderate levels of AD on the EPCCE (cited above as a measure of multidimensional constructs that overlap with judgment), a 32-item measure of problem solving related to finances, medications, transportation, phone usage, household and meal preparation. The participant is shown 16 stimuli and asked to solve 2 problems related to each target. The patients were divided into a group with moderate cognitive impairment (score 11 to 19 on MMSE) and another with Mild Cognitive Impairment (score 20 to 23 on MMSE). Scores on the EPCCE differed significantly between the groups, with the less impaired subjects demonstrating better performance.

Regarding specific judgment tests, four studies were found, describing the performance of controls and patients with dementia on the TOP-J, Problem Solving Sub-scale of the ILS and NCSE JQ judgment tests (Table 1). The NCSE JQ was not able to differentiate between controls and less impaired patients^8^ and two other studies^5,36^ confirmed the instrument was unable to differentiate even controls from more impaired patients. The Problem Solving Sub-scale of the ILS was not able to differentiate cognitive impairment no dementia from mild dementia patients, but differentiated these groups from normal cognitive status and moderate dementia groups.^50^ The TOP-J showed similar performance for MCI and subjects with normal cognition with complaints (CC). However, it seems to be a reliable measure to distinguish these groups from subjects with normal cognition without complaints and AD patients.^5^

No studies regarding the NAB JDC judgment test in AD patients were found. Similarly, no reports regarding judgment measures in other specific dementias were retrieved.

## Conclusion

Judgment is intimately linked with the process of problem solving and decision making, and these terms are often used interchangeably in the neuropsychological literature. Nevertheless, there are important practical differences between these processes.

Judgment in everyday situations is an important aspect that should be incorporated into the neuropsychological assessments of older people. However, despite its importance, there is a lack of studies focusing on judgment measures.

Additional studies on judgment involving subjects with various degrees of cognitive impairment, using more ecological and specific measures may be useful to support clinicians' inferences about how patients are able to live independently in a safe manner and also to support decisions by other professionals in juridical interventions. Currently, our group is working on an adapted version of the TOP-J for Brazilian samples of cognitively healthy older adults, Mild Cognitive Impairment and dementia patients.

Although clinical opinion is currently an accepted standard to determine everyday competence, the lack of a gold standard casts doubt on clinical judgments.
